# Association between Poor Appetite and Nutrient Intake in Community-Dwelling Older Adults: The Otassha Study

**DOI:** 10.31662/jmaj.2025-0467

**Published:** 2026-03-06

**Authors:** Yurie Mikami, Keiko Motokawa, Maki Shirobe, Masanori Iwasaki, Tatsunosuke Gomi, Misato Hayakawa, Lena Käläntär, Yasuyuki Sakata, Yutaka Watanabe, Hisashi Kawai, Shuichi Obuchi, Yoshinori Fujiwara, Kazushige Ihara, Hirohiko Hirano

**Affiliations:** 1 Tokyo Metropolitan Institute for Geriatrics and Gerontology, Tokyo, Japan; 2 Faculty of Dental Medicine and Graduate School of Dental Medicine, Hokkaido University, Hokkaido, Japan; 3 Health Care and Nutritional Science Institute, Morinaga Milk Industry Co., Ltd., Kanagawa, Japan; 4 Department of Oral Health Science, Hokkaido University, Hokkaido, Japan; 5 Department of Social Medicine, Hirosaki University Graduate School of Medicine, Aomori, Japan

**Keywords:** appetite, BDHQ, community-dwelling, nutrient intake, older adults, SNAQ

## Abstract

**Introduction:**

Poor appetite is a prevalent and serious problem among older adults. However, real-world data regarding appetite and nutrient intake among community-dwelling older adults are scarce. The aim of this study was to clarify the association between appetite and nutrient intake status among community-dwelling older adults.

**Methods:**

This cross-sectional study involved participants (N = 583) from the cohort of the “Otassha study,” conducted among community-dwelling older adults. Appetite was assessed using the Japanese version of the Simplified Nutritional Appetite Questionnaire. Nutrient intake was calculated using the Brief-Type Self-Administered Diet History Questionnaire. In addition, compliance status was assessed using the recommended dietary allowance (RDA) of various nutrients based on the “Dietary Reference Intakes for Japanese (2020).” The association between compliance status and appetite was analyzed using logistic regression, with the RDA of multiple nutrients as the dependent variable and poor appetite as the independent variable.

**Results:**

In the study population, 32.1% of participants had poor appetite. Multivariable logistic regression revealed that poor appetite was significantly associated with insufficient vitamin C intake relative to the RDA (odds ratio 1.67; 95% confidence interval 1.08-2.57).

**Conclusions:**

This study demonstrated an association between poor appetite and vitamin C intake among community-dwelling older adults.

## Introduction

Poor appetite is common among older adults, with a prevalence of approximately 20%-37% ^(1), (2), (3), (4), (5), (6)^. The causes of poor appetite include taste and smell alterations and a decrease in the secretion of appetite-regulating hormones ^(7), (8)^. In addition to these physiological changes associated with aging, other factors contribute to poor appetite in older adults. These include physical and mental factors, such as depression; medication effects; diseases such as gastrointestinal diseases, chronic obstructive pulmonary disease, Parkinson’s disease, and heart disease; gastrointestinal malabsorption; reduced physical activity; and diminished masticatory function. Environmental factors, including living alone, social isolation, institutionalization, economic distress, and monotonous eating habits, also contribute to poor appetite ^(7), (8), (9)^. Additionally, physiological, physical, mental, and environmental factors are likely intricately intertwined, contributing to poor appetite. These, in turn, lead to reduced dietary intake and weight loss, ultimately resulting in undernutrition.

The Dietary Reference Intakes for Japanese (2020) establishes standards for energy and nutrient intakes necessary for maintaining and promoting health and for preventing lifestyle-related diseases ^(10)^. Since the 2020 edition, the standards also consider the prevention of undernutrition and frailty in older adults.

To prevent nutritional deficiencies, it is necessary to avoid negative nutritional practices, such as insufficient nutrient and energy intake. The Dietary Reference Intakes for Japanese established the estimated average requirement (EAR), recommended dietary allowance (RDA), and adequate intake as benchmarks to help prevent inadequate intakes ^(10)^. The EAR, based on robust evidence, represents the intake level that meets the needs of 50% of the population. The RDA is set for nutrients with established EARs and is defined as the amount sufficient for 97%-98% of the population. Conversely, adequate intake is determined when there is insufficient evidence to calculate the EAR and aims to maintain a specific nutritional status. The goal of these indicators is to prevent insufficient nutrient and energy intakes. Appetite influences dietary intake, and poor appetite often leads to decreased nutrient and energy consumption. Notably, undernutrition is a serious prognostic issue in older adults ^(11), (12)^. According to Fried et al. ^(13)^, poor appetite is part of the frailty cycle and may accelerate its progression. Thus, improved appetite may contribute to maintaining nutritional status.

However, information on poor appetite and nutrient intake in older adults is limited, and the relationship between appetite and nutrient intake based on the Dietary Reference Intakes for Japanese among community-dwelling older adults has not been assessed. Therefore, the present study aimed to elucidate the relationship between appetite and nutrient intake status in community-dwelling older adults.

## Materials and Methods

### Participants

This cross-sectional study was part of a cohort study, the “Otassha study,” which has been conducted since 2011 among community-dwelling older adults living in Itabashi Ward, Tokyo, Japan. Details of the study have been previously described ^(14)^. In the present study, based on the basic resident register, questionnaires were mailed to adults aged ≥65 years in the surrounding areas of the Tokyo Metropolitan Institute for Geriatrics and Gerontology, and 2,292 participants were recruited. Among the 642 participants who took part in the survey from September to October 2021, 583 participants with no history of depression and no missing items in the dietary survey were selected for analysis.

### Survey items

#### Appetite

Appetite was assessed using the Japanese version of the Simplified Nutritional Appetite Questionnaire (SNAQ) ^(15), (16)^. The participants were evaluated on a five-point scale across four items: appetite, feeling of fullness, taste of food, and daily meal frequency. Scores were calculated based on their total points. Poor appetite was defined as having a SNAQ score of ≤14 ^(16)^.

#### Nutrient and energy intakes

Nutrient and energy intakes were assessed using the Brief-Type self-administered Diet History Questionnaire (BDHQ) ^(17)^, a self-administered questionnaire in which participants report their dietary habits in the past month. The participants completed the BDHQ to estimate nutrient intakes. The BDHQ assesses dietary intake in five categories: (1) intake frequency of food and non-alcoholic beverages; (2) daily intake of rice (including type) and miso soup; (3) frequency of drinking alcoholic beverages and amount per drink for five alcoholic beverages; (4) usual cooking methods; and (5) general dietary behavior. Subsequently, trained dietitians interviewed the participants.

Using the BDHQ nutrient intake results, compliance status was evaluated using the RDA for protein, calcium, iron, vitamin A, vitamin B_1_, vitamin B_2_, and vitamin C based on Dietary Reference Intakes for Japanese 2020 ^(10)^.

#### Other items

Sex, age, and medical history―including hypertension, stroke, heart disease, diabetes mellitus, dyslipidemia, osteoporosis, chronic renal failure, pneumonia, chronic obstructive pulmonary disease, osteoarthritis, spinal stenosis, fractures, malignant neoplasms, depression, autoimmune diseases, and ulcerative colitis―were assessed. The number of items in each participant’s medical history (calculated from the above conditions), medications taken (0-4 or ≥5 types), cognitive status assessed using Mini-Mental State Examination (MMSE) scores, and residential status (living alone or not) were also evaluated.

Height and weight were measured, and body mass index was calculated as weight (kg) / {height (m) × height (m)}.

### Statistical analysis

Differences in continuous and categorical variables between the groups with and without poor appetite were analyzed using the Mann-Whitney *U* test or χ^2^ test, as appropriate. Subsequently, the association between compliance status with RDA for various nutrients (dependent variable) and poor appetite (independent variable) was assessed using logistic regression analyses. Adjustment variables included factors previously reported to be associated with appetite. In Model 1, these variables included sex, age, number of medications taken, number of relevant medical histories, MMSE score, and living situation, whereas in Model 2, variables for Model 1 and energy intake were included ^(5), (7), (8)^. In the present study, in Model 2, only energy intake was added to avoid overadjustment, as the other nutrients were already adjusted for. All statistical analyses were performed using IBM SPSS Statistics for Windows, version 26 (IBM Corp., Armonk, NY, USA). Results with p < 0.05 were considered statistically significant.

### Ethical statements

This study was approved by the Ethical Review of the Research Department of the Tokyo Metropolitan Healthy Longevity Medical Center (No. R21-056). Informed consent was obtained from all participants.

## Results

Overall, 32.1% of the participants had poor appetite. These participants were significantly older and exhibited lower body mass index, SNAQ score, energy intake, and MMSE score than participants without poor appetite. Furthermore, significant differences were observed in the rate of living alone, which was higher in the group with poor appetite than in the group without poor appetite (Table 1).

**Table 1.** Patient Characteristics in The Two Groups.

		With poor appetite (n = 187)		Without poor appetite (n = 396)		p-Value
		n / Mean	% / SD	n / Mean	% / SD	
Sex*	Male	71	(38.0)	157	(39.6)	0.717
	Female	116	(62.0)	239	(60.4)	
Age^†^	(years)	74.6 ± 6.7		73.3 ± 6.6		0.029
BMI^†^	(kg/m^2^)	22.3 ± 3.6		23.3 ± 3.4		<0.001
SNAQ score^†^	(point)	13.3 ± 0.9		15.9 ± 0.9		<0.001
Energy intake^†^	(kcal)	1839.9 ± 622.1		1976.8 ± 587.2		0.002
Medical history*	Number^†^	2.1 ± 1.5		2.0 ± 1.5		0.282
	Hypertension	84	(44.9)	167	(42.2)	0.296
	Stroke	7	(3.7)	22	(5.6)	0.233
	Heart disease	34	(18.2)	77	(19.4)	0.405
	Diabetes mellitus	29	(15.5)	43	(10.9)	0.076
	Dyslipidemia	72	(38.7)	163	(41.2)	0.319
	Osteoporosis	35	(18.7)	56	(14.1)	0.098
	Chronic renal failure	2	(1.1)	4	(1.0)	0.625
	Pneumonia	4	(2.1)	9	(2.3)	0.591
	Chronic obstructive pulmonary disease	5	(2.7)	5	(1.3)	0.186
	Osteoarthritis	28	(15.0)	60	(15.2)	0.531
	Spinal stenosis	17	(9.1)	53	(13.4)	0.086
	Fractures	33	(17.6)	56	(14.1)	0.165
	Malignant neoplasms	36	(19.3)	59	(14.9)	0.114
	Autoimmune diseases	11	(5.9)	16	(4.0)	0.216
	Ulcerative colitis	1	(0.5)	0	(0.0)	0.321
Number of medications*	0-4	125	(66.8)	289	(73.0)	0.143
	5 or more	62	(33.2)	107	(27.0)	
MMSE score^†^	(Point)	27.3 ± 2.8		28.2 ± 1.9		<0.001
Residential status*	Living alone	59	(31.6)	82	(20.7)	0.005

*χ^2^ test. ^†^Mann-Whitney *U* test. BMI: body mass index; MMSE: Mini-Mental State Examination; SD: standard deviation; SNAQ: Simplified Nutritional Appetite Questionnaire.

Based on the Dietary Reference Intakes for Japanese 2020, compliance status with the RDA for various nutrients was compared between groups with and without poor appetite. The poor appetite group demonstrated a significantly lower rate of sufficient intake of calcium, iron, vitamin A, vitamin B1, vitamin B2, and vitamin C than the group without poor appetite (Table 2).

**Table 2.** Comparison of the Rate of Participants with Insufficient RDA Between the Groups.

	With poor appetite		Without poor appetite		p-Value
	n	%	n	%	
Protein	36	19.3	58	14.6	0.184
Calcium	116	62.0	195	49.2	0.004
Iron	51	27.3	72	18.2	0.016
Vitamin A	95	50.8	157	39.6	0.012
Vitamin B_1_	148	79.1	276	69.7	0.017
Vitamin B_2_	72	38.5	101	5.5	0.002
Vitamin C	74	39.6	104	26.3	0.001

χ^2^- test. “insufficient” means below RDA. RDA: recommended dietary allowance.

Multivariable logistic regression analyses revealed that poor appetite was significantly associated with insufficient intake of calcium (odds ratio [OR] 1.79; 95% confidence interval [CI] 1.23-2.62), iron (OR 1.66; 95% CI 1.07-2.57), vitamin A (OR 1.49; 95% CI 1.03-2.16), vitamin B1 (OR 1.87; 95% CI 1.20-2.93), vitamin B2 (OR 1.98; 95% CI 1.32-2.96), and vitamin C (OR 1.98; 95% CI 1.33-2.94) relative to the RDA in Model 1. However, in Model 2, which incorporated energy intake, only insufficient intake of vitamin C relative to the RDA was associated with poor appetite (OR 1.67; 95% CI 1.08-2.57) (Table 3).

**Table 3.** Binomial Logistic Regression Analysis with Various Nutrients as Dependent Variables.

	Crude			Model 1			Model 2		
	Odds ratio	95% Confidence interval		Odds ratio	95% Confidence interval		Odds ratio	95% Confidence interval	
Protein	1.39	0.88-2.20		1.25	0.77-2.03		0.77	0.40-1.50	
Calcium	1.68	1.18-2.40	*	1.79	1.23-2.62	*	1.44	0.90-2.31	
Iron	1.69	1.12-2.54	*	1.66	1.07-2.57	*	1.25	0.74-2.13	
Vitamin A	1.57	1.11-2.23	*	1.49	1.03-2.16	*	1.17	0.77-1.79	
Vitamin B_1_	1.65	1.09-2.49	*	1.87	1.20-2.93	*	1.72	0.91-3.25	
Vitamin B_2_	1.83	1.26-2.65	*	1.98	1.32-2.96	†	1.57	0.97-2.57	
Vitamin C	1.84	1.27-2.66	*	1.98	1.33-2.94	†	1.67	1.08-2.57	*

Binomial logistic regression; independent variable: SNAQ Score (0: without poor appetite, 1: with poor appetite), dependent variable: compliance status of protein, calcium, iron, vitamin A, vitamin B_1_, vitamin B_2_, vitamin C (0: sufficient, 1: insufficient). *p < 0.05, †p < 0.001. Model 1: sex, age, number of medical histories, number of medications, MMSE score, living alone; Model 2: Model 1 + energy intake. MMSE: Mini-Mental State Examination; SNAQ: Simplified Nutritional Appetite Questionnaire.

## Discussion

Although previous research has explored the relationship between various nutrient intakes and appetite, the present study specifically focused on adherence to RDA standards. The RDA represents the level of intake adequate to prevent nutrient deficiencies in most individuals within a population, and nutrient consumption should ideally meet this standard. The present study revealed that participants with poor appetite exhibited lower overall dietary intake than those with normal appetite. Among the nutrients for which the RDA has been set (protein, calcium, iron, vitamin A, vitamin B1, vitamin B2, and vitamin C), RDA deficiency of minerals and vitamins was associated with poor appetite (Model 1; Table 3). Previous studies have indicated that poor appetite is associated with low food intake ^(5)^ and limited dietary variety ^(1)^, potentially leading to inadequate nutrient intake ^(2), (18)^. Moreover, enhanced dietary variety reportedly boosts overall dietary intake ^(19), (20)^. Thus, it is important to promote increased total dietary intake for individuals with poor appetite and to improve dietary variety to ensure comprehensive nutrient intake.

In the present study, in Model 2, which incorporated energy intake as a covariate, vitamin C intake was associated with poor appetite among the nutrients for which the RDA has been established. It is important to prevent frailty among older adults. Poor appetite is considered an accelerator of frailty, and depression is associated with the background of poor appetite ^(7), (8)^. On the other hand, in mice, low blood vitamin C levels are reported to increase symptoms of depression and anxiety, leading to poor appetite ^(21)^. Similarly, in humans, insufficient vitamin C intake may exacerbate this condition, leading to a detrimental cycle. A previous study reported that blood vitamin C levels were associated with frailty severity ^(22), (23)^. Therefore, it is important to ensure sufficient vitamin C intake. While numerous fruits and vegetables are rich in vitamin C, individuals with poor appetites often consume fewer of these foods ^(1), (5)^. In other words, poor appetite can lead to decreased vitamin C intake due to reduced consumption of fruits and vegetables. Consistent with this notion, vitamin C intake is important for improving poor appetite; moreover, dietary variety, such as incorporating vegetables and fruits, is recommended. However, the intake of fruits and vegetables in Japan falls short of the recommended amounts ^(24)^, likely owing to financial constraints and lack of access to these foods ^(25)^.

In the present study, 32.1% of the participants had poor appetite. These participants were significantly older than participants in the group without poor appetite. Although poor appetite has been reported to increase with age among older adults ^(5), (6), (8)^, its prevalence in the present study was higher than that in previous studies. This difference might be due to different methods of evaluating appetite. The evaluation of appetite varies widely, ranging from objective assessments of diminished dietary intake to subjective evaluations, such as the presence of appetite measured using visual analog and Likert scales, as well as quantitative measures through questionnaires, such as the Functional Assessment of Anorexia/Cachexia Therapy and SNAQ. In the present study, appetite was assessed using the SNAQ, which can be administered by non-specialists. The SNAQ quantifies and compositely evaluates appetite, including the feeling of fullness, food taste, meal frequency, and appetite ^(16), (17), (26)^. Therefore, the prevalence of poor appetite assessed using the SNAQ might be higher than that assessed using single-factor evaluations. While assessing appetite by decreased dietary intake is relatively simple, self-monitoring by community-dwelling individuals to determine changes in their dietary intake remains difficult. Poor appetite is affected not only by age-related changes but also by various other factors, with causes differing among individuals. Consequently, understanding these causes and providing appropriate support might improve appetite, thereby aiding in the prevention of undernutrition and frailty.

The present study has certain limitations. First, it primarily targeted older adults residing in a single urban area, which may limit the generalizability of the findings to rural or low-income populations. Second, this study was cross-sectional; therefore, the findings should be interpreted with caution. Third, nutrient and energy intakes were calculated using the BDHQ and may have been influenced by participants’ memory recall. However, this effect appears to be minimal, as the analysis was also adjusted for cognitive function. Moreover, accurate nutrient intakes cannot be definitively determined using the BDHQ because of the possibility of under- or over-reporting. Nevertheless, the BDHQ is a useful evaluation tool for large-scale surveys because it is less burdensome for participants. Finally, although the BDHQ collects information on the use of nutritional supplements, detailed data were not obtained.

In conclusion, this study demonstrated an association between poor appetite and vitamin C intake status among community-dwelling older adults. Poor appetite may serve as a critical nutritional factor in countermeasures against frailty. Future longitudinal studies or interventions focusing on appetite, with the aim of facilitating social implementation, are required.

## Article Information

### Acknowledgments

The authors thank the involved staff and participants. We would also like to thank Editage (www.editage.jp) for English language editing.

### Author Contributions

Data curation: Keiko Motokawa, Maki Shirobe, Masanori Iwasaki, Hisashi Kawai. Formal analysis: Yurie Mikami. Funding acquisition: Keiko Motokawa, Shuichi Obuchi. Investigation: Yurie Mikami, Keiko Motokawa, Maki Shirobe, Masanori Iwasaki, Misato Hayakawa, Lena Käläntär. Project administration: Keiko Motokawa, Maki Shirobe, Masanori Iwasaki, Hisashi Kawai., Shuichi Obuchi, Hirohiko Hirano. Supervision: Shuichi Obuchi, Hirohiko Hirano. Writing: Yurie Mikami. Writing - review & editing: Keiko Motokawa, Maki Shirobe, Masanori Iwasaki, Tatsunosuke Gomi, Misato Hayakawa, Lena Käläntär, Yasuyuki Sakata, Yutaka Watanabe, Hisashi Kawai., Shuichi Obuchi, Yoshinori Fujiwara, Kazushige Ihara, Hirohiko Hirano.

### Conflicts of Interest

This is a collaborative study with Morinaga Milk Industry Co., Ltd. The principal investigator for this collaborative study was Keiko Motokawa. Yasuyuki Sakata is an employee of Morinaga Milk Industry Co., Ltd.

### IRB Approval Code and Name of the Institution

This study was approved by the Ethical Review of the Research Department of the Tokyo Metropolitan Healthy Longevity Medical Center (No. R21-056). Informed consent was obtained from all participants.
